# Electrolyte Effects on Electrochemical CO_2_ Reduction Reaction at Sn Metallic Electrode

**DOI:** 10.1021/acs.jpcc.4c06361

**Published:** 2024-12-05

**Authors:** Maria Rodrigues Pinto, Rafaël E. Vos, Raphael Nagao, Marc T. M. Koper

**Affiliations:** † Institute of Chemistry, University of Campinas, Campinas, São Paulo 13083-970, Brazil; ‡ Leiden Institute of Chemistry, Leiden University, P.O. Box 9502, Leiden 2300 RA, The Netherlands; § Center for Innovation on New Energies, University of Campinas, Campinas, São Paulo 13083-841, Brazil

## Abstract

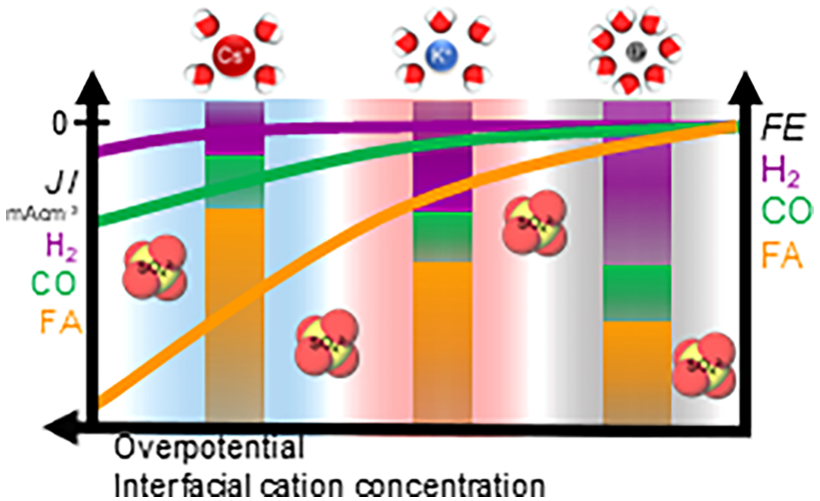

Understanding the electrolyte factors governing the electrochemical CO_2_ reduction reaction (CO_2_RR) is fundamental for selecting the optimized electrolyte conditions for practical applications. While noble metals are frequently studied, the electrolyte effects on the CO_2_RR on Sn catalysts are not well explored. Here, we studied the electrolyte effect on Sn metallic electrodes, investigating the impact of electrolyte concentration, cation identity, and anion properties, and how it shapes the CO_2_RR activity and selectivity. The activity for formic acid and carbon monoxide increases with the cation concentration and size at mild acid conditions. In contrast, hydrogen production is not strongly influenced by the cathodic potential, electrolyte concentration, and cation size. Furthermore, we have compared the CO_2_RR performance at a constant cation concentration in K_2_SO_4_ (pH 4) and KHCO_3_ (pH 7), where we show that the reaction rate toward HCOOH and CO are minimally impacted by the anion identity on the SHE scale, while being affected by the cations in solution, which we attribute to the reaction being limited by cation-coupled electron transfer steps rather than by a proton-coupled electron transfer step. We propose that the HCOOH forms via adsorbed hydrides leading to *OCHO intermediate, while CO forms through an electron transfer step, producing *CO_2_^δ−^. Cations facilitate both processes by stabilizing the negatively charged intermediates, and the difference in the extent of the promotion of HCOOH over CO formation would stem from the stronger cation effects on *H compared with *CO_2_^δ−^ species. Additionally, the presence of HCO_3_^–^ at high concentrations (1.0 mol L^–1^) is shown to significantly enhance the production of H_2_ at high overpotentials (>-1.0 V vs RHE) due to bicarbonate ions acting as protons donors, outcompeting water reduction. These findings underscore the significance of electrolyte engineering for enhanced formic acid synthesis, offering valuable insights for optimizing the CO_2_RR processes on Sn electrocatalysts.

## 1 Introduction

The electrochemical CO_2_ reduction reaction (CO_2_RR) when combined with renewable electricity sources constitutes an alternative to fossil-resource technologies, by which CO_2_ is converted into low-carbon footprint fuels and valuable chemicals.^1^ The CO_2_RR consists of a series of proton-coupled electron transfer steps, through which different products can be obtained, including formic acid, carbon monoxide, hydrocarbons, and alcohols.^2,3^ Among the CO_2_RR-derived products, formic acid has many advantages in transportability and storage, being one of the most economically promising products.^2^ It is formed through a two-electron transfer reaction and can be obtained with high faradaic efficiencies on post-transition metals, such as Sn, Pb, In, and Bi.^3^

Several possible routes of CO_2_RR to HCOOH have been reported in the literature,^4−7^ and various studies have compared the binding energies of CO_2_RR intermediates to rationalize the selectivity for CO or HCOOH production among metallic catalysts. Bagger et al.^5^ showed that H* binding energy can be a good descriptor to differentiate between CO and HCOOH-producing metals. Feaster et al.^6^ compared the calculated binding energies of CO_2_RR intermediates with the partial current densities for HCOOH to show that the oxygen-bound intermediate *OCHO interacts more strongly with the Sn surface than the carbon-bound intermediate *COOH.^10^ The most widely accepted mechanism for the formation of HCOOH involves the formation of an *OCHO intermediate. In this mechanism, CO_2_ undergoes hydrogenation through the direct insertion of adsorbed hydride species from the catalyst or through the direct protonation by proton donor species in solution.

Among the formic acid-selective catalysts, Sn-based catalysts are attractive candidates for producing formic acid from CO_2_RR, given the relative abundance of Sn, its nontoxicity, and the high faradaic efficiencies for formic acid.^8^ However, achieving significant reaction rates requires high overpotentials, directly impacting production costs. To enhance the overall efficiency, we must explore strategies to modulate the performance of the CO_2_RR, aiming for more precise control over the electrochemical synthesis of HCOOH. The effect of various parameters on the electrochemical CO_2_RR at Sn-based electrodes have been reported in the literature, with a wide range of faradaic efficiencies, often attributed to differences in the electrode and/or electrolyte nature and the operating conditions.^8−12^

Most efforts to improve the CO_2_RR performance centered on modifying the catalyst properties and morphologies.^11^ However, the composition of the electrolyte substantially influences the CO_2_RR, offering an interesting strategy for fine-tuning the reaction activity and selectivity beyond catalyst modification.^13,14^ Interfacial reactions and intermediates depend on the local distribution of species dissolved in the electrolyte, such as CO_2_, HCO_3_^–^, OH^–^, and H^+^,^15,16^ which is influenced by the identity of both cations and anions of the electrolyte.^14^ Cations have been reported to be crucial for the formation of CO in the CO_2_RR on Au, Ag, and Cu electrodes,^17^ which has been ascribed to the key role of cations in stabilizing the negatively charged *CO_2_^δ−^ reaction intermediate. Concerning the formation of HCOOH, recent work from our group has studied the formation of HCOOH and *CO on a well-defined epitaxially grown Pd monolayer on Pt(111) and found that cations have a bigger impact on the formation of HCOOH, which is mediated by negatively charged hydride, than on *CO formation.^18^ Unlike Pd_ML_Pt(111), high overpotentials are needed to obtain significant HCOOH formation rates on Sn.^8^ This prompts the question of whether there are differences in the electrolyte effects at different applied potentials and different catalysts and whether the same intermediates are expected to impact activity and electrolyte effects.

As tin is an unstable metal that can oxidize in the atmosphere or under open circuit potential,^19^ in this study, we avoid the formation of oxides to investigate CO_2_RR performance on polycrystalline Sn. Specifically, we looked at the activity and selectivity for HCOOH, CO, and H_2_ with varying electrolyte concentrations and cation sizes in pH 4 environments. Performing CO_2_RR in mild acid electrolytes offers some advantages for upscaling technologies, HCO_3_^–^ crossover, and better compatibility with electrolyzer designs.^20,21^ From a more fundamental point of view, it offers the advantage of testing the effect of cations at very low cation concentrations in the absence of bicarbonate. Our results show that at bulk pH 4 the activity for HCOOH and CO formation increases with the electrolyte concentration and cation size, while there is not a strong effect on the formation of H_2_. We also discuss the role of HCO_3_^–^ acting as a proton donor, favoring HER in neutral media compared to pH 4.

## 2 Materials and Methods

The experiments were conducted in a homemade PEEK H-cell, with the details of the setup described elsewhere.^22^ The cell was cleaned in a permanganate solution overnight (0.5 mol L^–1^ H_2_SO_4_, 1 g L^–1^ KMnO_4_) and thoroughly rinsed and immersed in a diluted piranha acid solution to eliminate any remaining manganese oxides. Before use, the cell was rinsed and boiled in a Milli-Q water at least three times. The working electrode was a flat disc made of polycrystalline Sn (99.99% pure, Mateck) with a geometric surface area of 0.785 cm^2^. The working electrode was polished between experiments with sandpaper (SiC P4000, MicroCut Discs) and then with diamond polishing suspension of decreasing particle size (3.0, 1.0, and 0.25 μm, Buehler) on 8 in. micro polishing cloths. After polishing, the electrode was sonicated in ethanol and Milli-Q water for 3 min to remove any impurities and then dried with pressurized air.

The experiments were conducted using a three-electrode setup with a working and reference electrode (Hydroflex RHE—Gaskatel) in the same compartment. The catholyte compartment was separated from the counter electrode (dimensionally stable anode DSA, Magneto) with an ion exchange membrane (AMVN Selemion, AGC). Before beginning electrolysis, CO_2_ was purged in the electrolyte for 15 min and maintained throughout the experiment through a PEEK-frit (0.2 um pore size, IDEX) at 20 mL min^–1^ controlled by a mass flow controller (SLA5850, Brooks Instrument) from the bottom of the cell^22^ to increase mass transport in the cell. The working electrode was held at −0.2 V vs RHE to prevent Sn oxidation during electrolyte purging. 85% ohmic drop compensation was performed for all chronoamperometry measurements after determining ohmic resistance using Electrochemical Spectroscopy Impedance at −0.2 V vs RHE. The remaining 15% of ohmic drop was corrected after the experiments; all potentials reported here have been Ohmic drop-corrected accordingly. All electrochemical measurements were conducted using an IviumStat potentiostat.

Liquid products were measured using high-performance liquid chromatography (HPLC, Shimadzu), with an Aminex HPX-87H column (Biorad). The chromatograms from the standard samples and the corresponding calibration curve for formic acid quantification are provided in Figure S1. Gaseous products were analyzed online using a Shimadzu 2014 gas chromatograph with a TCD (with a Shincarbon column) and FID (with an RTX-1 column) detectors at 3 different times during the electrolysis. The selectivity was determined by combining the average of the 3 gaseous samples with HPLC data for each electrolysis experiment. During the electrolysis experiments, only H_2_ and CO gases were detected and formic acid was the only liquid product identified, where the sum of the FEs for every individual electrolysis experiment is between 80 and 106%. The values reported are averages of at least three repetitions with twice the standard deviation as the reported error bars.

The electrolytes were prepared from Li_2_SO_4_ (99.99% Suprapur, Sigma-Aldrich), K_2_SO_4_ (99.999% Suprapur, Sigma-Aldrich), Cs_2_SO_4_ (99.99% Thermo Scientific), and KHCO_3_ (99.95%, Sigma-Aldrich). Before each measurement was started, the electrolyte pH was adjusted using H_2_SO_4_ (95–98%, Sigma-Aldrich), after the solutions were saturated with CO_2_. H_2_SO_4_, H_2_O_2_ (35%, Merck), and KMnO_4_ (99%, Sigma-Aldrich) were used for the cleaning procedure.

## 3 Results

### 3.1 Effect of Electrolyte Concentration

**Figure 1:**
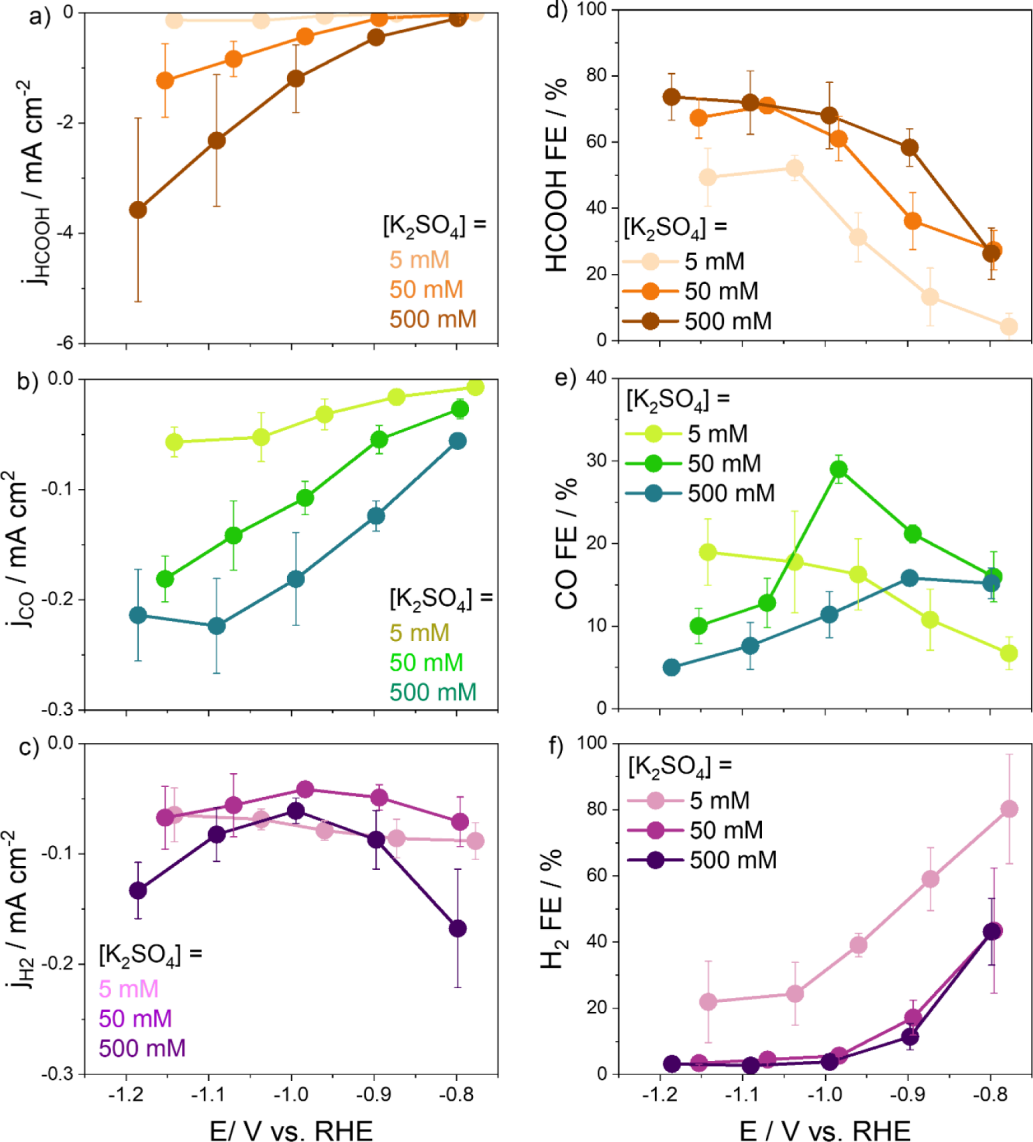
Activity and selectivity for Sn at different electrolytes K_2_SO_4_ concentrations of 5, 50, and 500 mmol L^–1^ at pH 4. Partial density currents (a-c) and faradaic efficiencies (d–e) for (a, d) formic acid, (b, e) carbon monoxide, and (c, f) hydrogen. The error bars represented in the figure are twice the standard deviation and are determined from at least three separate experiments. The lines are guides to the eye.

We studied the effect of the electrolyte concentration on CO_2_RR in mild acidic electrolytes on polycrystalline Sn between −0.8 to −1.2 V vs the reversible hydrogen electrode (RHE) by constant potential electrolysis at pH 4 and x mmol L^–1^ of K_2_SO_4_, with x = 5, 50, and 500, employing the H-cell setup, as shown in Figure S2. As reported in the literature, CO_2_RR on Sn primarily leads to the formation of HCOOH, but often CO is reported as a minority product, along with H_2_ as a result of the competing HER.^11^Figure 1 shows the performance of the polycrystalline metallic Sn electrode evaluated at pH 4 and different K_2_SO_4_ concentrations. We evaluated the partial current density for HCOOH (j_HCOOH_), CO (j_CO_), and H_2_ (j_H2_) (Figure 1a,b and c, respectively) and associated Faradaic efficiencies (FE) (Figure 1d,e and f). The partial current density for HCOOH and CO production increases with increasing both overpotential and [K_2_SO_4_] (Figure 1a,b), in agreement with literature.^12,23,24^ Similar to what has been observed for Pd_ML_Pt(111) at pH 3, CO_2_RR on Sn is cation-promoted, with a more notable influence on the formation of HCOOH than on CO.^18^ However, unlike Pd, this effect on Sn is not confined to low overpotentials, as Sn exhibits weak CO adsorption, thereby preventing CO poisoning.^25,26^

The electrolyte concentration has a positive effect on both activity and selectivity for HCOOH reflected in the values for j_HCOOH_ (Figure 1a) and FE(HCOOH) (Figure 1d). The j_HCOOH_ increases as the potential decreases from −0.8 to −1.2 V vs RHE, while the FE(HCOOH) increases until −1.0 V vs RHE, where it reaches the maximum around 70% of FE(HCOOH) between −1.0 and −1.2 V vs RHE for all three studied cation concentrations. The promotion of CO_2_RR under these conditions might be related to the effect of the cation concentration near the electrode surface, i.e., by stabilizing CO_2_RR intermediates or as a consequence of cation-induced differences in the electric field at the electric double layer.^17,27^

At high overpotentials (−1.2 V vs RHE) and high electrolyte concentrations (500 mmol L^–1^), the activities for HCOOH production are significantly higher than those for CO (j_HCOOH_ = −3.6 mA cm^–2^ and j_CO_ = −0.23 mA cm^–2^), which shows that HCOOH is preferentially formed, with CO being a minor product under these conditions. Under these more extreme conditions, the increased currents lead to greater current instabilities, as seen in Figure S2c, resulting in a larger variation in partial current density for HCOOH. Nevertheless, the reaction’s selectivity remains consistent, as supported by the error bars associated with the faradaic efficiency values for the formic acid production, as seen in Figure 1d. However, at low overpotential (>−1.0 V vs RHE) and [K_2_SO_4_] = 5 mmol L^–1^, the HER is the predominant reaction and the rates of HCOOH and CO production are comparable, as indicated in Figure S3, which highlights the significant impact of the potential and electrolyte conditions on the catalyst performance. Figure 1e shows distinct trends in the FE toward CO for each concentration. The FE(CO) falls within 5–30% for every concentration and applied potential. For the lowest concentration, i.e., [K_2_SO_4_] = 5 mmol L^–1^, the FE(CO) increases with rising overpotential. At the intermediate concentration, a volcano-like trend takes form, with the maximum FE(CO) reaching 30% at −1.0 V vs RHE. Finally, at [K_2_SO_4_] = 500 mmol L^–1^, the FE(CO) decreases from 15% to 5% with increasing overpotential. Often overlooked, the formation of CO on Sn can indeed constitute a significant portion of the product spectrum under certain conditions, particularly at low electrolyte concentrations, achieving FE(CO) of around 20%. It is essential to emphasize that to attain high FE(HCOOH) and substantial partial current densities, a significant electrolyte/cation concentration is required, along with the application of high overpotentials.

Unlike those of j_HCOOH_ and j_CO_, the j_H2_ values (Figure 1c) remain relatively unaffected by changes in potential and electrolyte concentration within the examined range. In fact, there is no discernible trend between j_H2_ and the applied potential or electrolyte concentration. Similarly, Resasco et al.^28^ observed for Cu(100) that the concentration of larger cations has a pronounced promotional effect on the formate partial current density, without a significant impact on H_2_ and CO production. By contrast, on Au electrodes, at constant HCO_3_^–^ concentration, an increase in cation concentration enhances the partial current density to CO and, more prominently, H_2_ formation.^29^ Thus, the variations in the reactivity depend strongly on the nature of the catalyst.

At low overpotentials, the Sn electrode exhibits a preference for the HER. The FE for H_2_ (Figure 1f) decreases with increasing overpotential in contrast with the FE(HCOOH), which increases as the potential becomes more negative, in agreement with the results of Feaster et al.^6^ For more negative potentials, the decrease in FE(H_2_) may be attributed to the buildup of the interfacial pH by increasing total current density, which should also impact the local cation concentration.^15,30^ The decrease in FE(H_2_) may also be rationalized in terms of the consumption of hydrides for the formation of HCOOH, which happens alongside the increase of CO_2_RR rates to HCOOH, as surface polarization has been proposed as a crucial trigger for hydride transfer.^31^

### 3.2 Effect of Cation Identity

**Figure 2:**
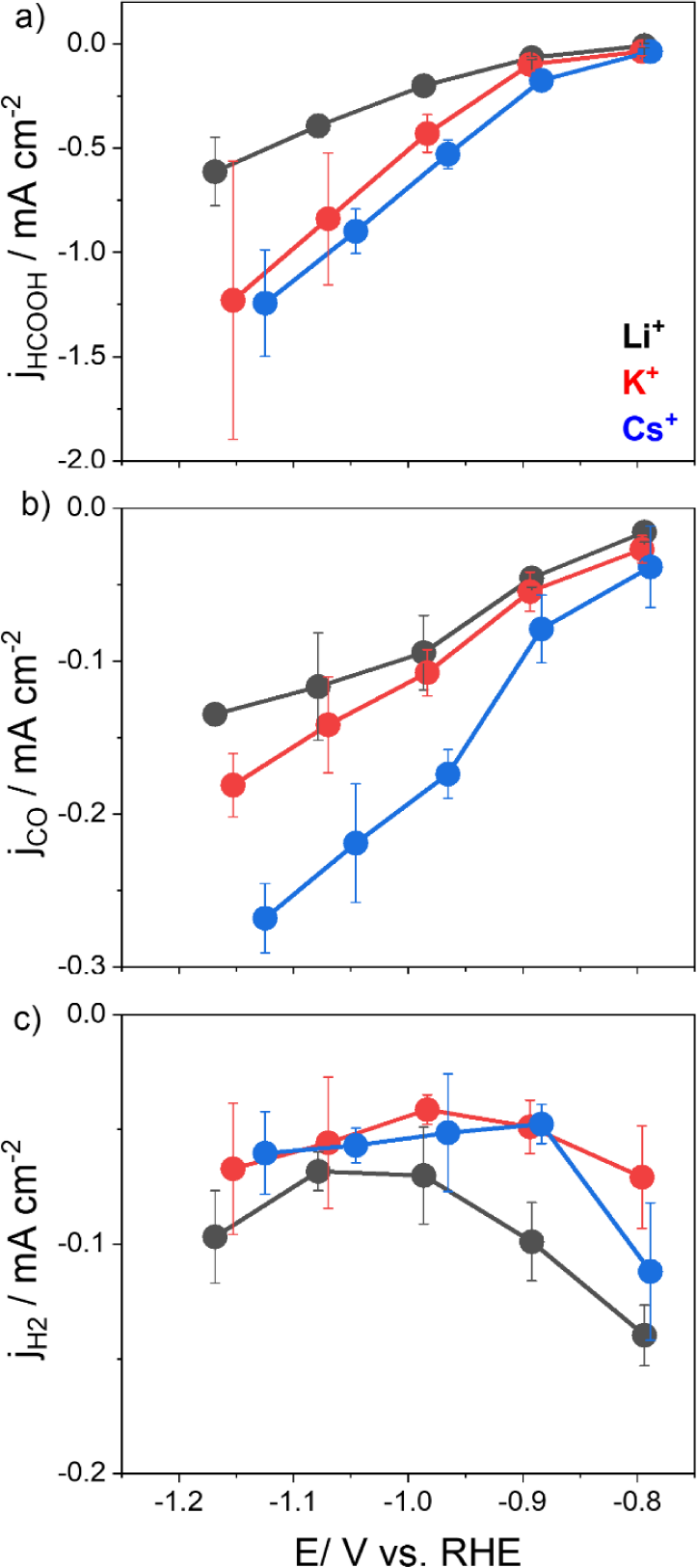
Activity of Sn for different products at pH 4 with different alkali sulfates, namely Li^+^ (black), K^+^ (red), and Cs^+^ (blue) at 50 mmol L^–1^. Partial density currents for (a) formic acid, (b) carbon monoxide, and (c) hydrogen. The error bars represented in the figure are twice the standard deviation, which are determined from at least three separate experiments. The lines are a guide to the eye.

We studied the effect of the identity of the alkali cation on the CO_2_RR on metallic Sn, as a function of potential at pH 4 and 50 mmol L^–1^ M_2_SO_4_, with M^+^ = Li^+^, K^+,^ and Cs^+^. Figure 2 shows the obtained partial current densities for HCOOH, CO, and H_2_ (Figure 2a–c) for the electrolysis experiments under constant potential in Figures S2b and S4. The corresponding FEs are displayed in Figure S5. Both the j_HCOOH_ and j_CO_ increase in the order Li^+^ < K^+^ < Cs^+^ but to a different extent. We observe a maximum j_HCOOH_ of −0.6 mA cm^–2^ at −1.2 V vs RHE (Figure 2a) for Li^+^, which is lower than the current densities obtained with the electrolyte containing K^+^ and Cs^+^. Moreover, the same trend applies to CO formation at these conditions, which is higher in solutions containing more weakly hydrated cations, following the ability of these species to accumulate near the surface.^17,32^ The faradaic efficiency for the HCOOH formation increases with overpotential for the experiments performed in all three cation electrolytes, with the highest FE(HCOOH) of 72% attained at −1.0 vs RHE in the K^+^ containing sulfate electrolyte. We did not detect clear trends and very major differences in FE for HCOOH and CO upon changing the cation identity (Figure S4a,b) in our experimental setup. However, we observed that K^+^ outperforms Cs^+^ and Li^+^ for FE(HCOOH) at all potentials studied, though the exact reason for this remains unclear. Therefore, at this point, the best we can do is compare Li^+^ to K^+^ or a strongly solvated cation to a weakly solvated cation. While K^+^ and Cs^+^ are weakly solvated cations,^13^ resulting in similar behavior in terms of partial current densities for the formation of HCOOH and CO (Figure 2a,b), Li^+^ is known as a strongly hydrated cation.^32^ We observed a generally lower performance for the CO_2_RR in Li^+^-containing sulfate electrolyte at the potential range studied for HCOOH formation in terms of faradaic efficiency and partial current density. At the same time, Li^+^ in solution seems to favor the HER compared to the experiments performed in the presence of K^+^ and Cs^+^ cations (Figures 2c and S4c). Generally, a strongly hydrated cation like Li^+^ has a lower effect on CO_2_RR than weakly hydrated cations, such as K^+^ and Cs^+^.^29^ These observations are in line with trends documented in previous literature,^12,33^ for Sn but also for a range of other metallic electrodes.^13,14,18,34^

There are three main theories described in the literature to explain the role of cations in the activity for CO_2_RR: through the modification of the local electric field,^35^ by buffering the interfacial pH,^30,36^ or through the stabilization of important CO_2_RR intermediates.^28,35^ The current prevailing mechanism involves stabilization of the *CO_2_^δ−^ intermediate by (hydrated) cations. This interaction has been demonstrated to positively impact CO_2_RR on Au and Cu electrodes, supported by both experimental and theoretical evidence, highlighting the electrostatic interaction induced by solvated cations in the outer Helmholtz plane.^28,29^

We observe that j_H2_ is not highly impacted by the cation size, as can be observed in Figure 2c. Resasco et al.^28^ found the same trend for Sn in HCO_3_^–^ electrolyte in neutral media at −1.0 V vs RHE, which was attributed to the fact that hydrogen adsorption is not affected by cations in solutions, which seems to be valid for pH 4 as well. Li^+^ electrolyte leads to a higher selectivity for HER, compared to K^+^ and Cs^+^, weakly hydrated cations (Figure S5c). The large hydration radius of Li^+^ hinders their accumulation at the outer Helmholtz plane (OHP), which agrees with the higher FE(H_2_) at a low cation concentration, discussed in the preceding section.

### 3.3 Mild Acid Media Compared to Neutral Bicarbonate Electrolyte

**Figure 3:**
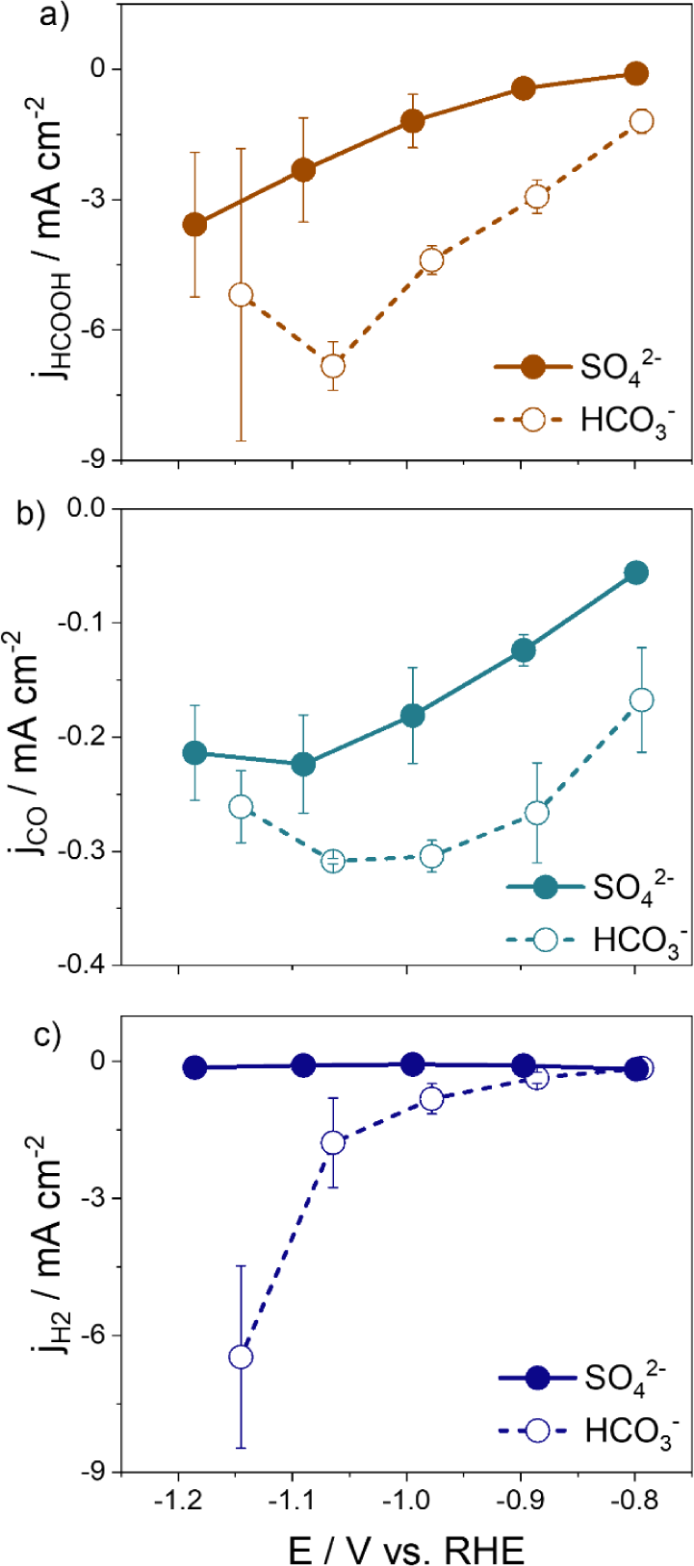
Activity for Sn comparing the effect of sulfate vs bicarbonate. [K^+^] = 1.0 mol L^–1^ for K_2_SO_4_ 0.5 mol L^–1^ with pH = 4 (solid line) and KHCO_3_ 1.0 mol L^–1^ at pH = 7 (dashed line). Partial density currents for (a) formic acid, (b) carbon monoxide, and (c) hydrogen. The error bars represented in the figure are twice the standard deviation, which are determined from at least three separate experiments. The lines are a guide to the eye.

To investigate if the effects observed in mildly acidic media with pH 4 in sulfate solutions are applicable in the more common bicarbonate solution with a pH of 7, we analyzed the partial current densities in both sulfate and bicarbonate solutions, while maintaining a constant K^+^ concentration. Figure 3 displays the potential dependence of the partial current densities for HCOOH, CO, and H_2_ (Figure 3a,b and c respectively) for the electrolysis experiments, with the chronoamperometry curves displayed in Figures S2c and S6b. The measurements were carried out in [K^+^] = 1.0 mol L^–1^ in SO_4_^2–^ (pH 4) and HCO_3_^–^ (pH 7). Note that the results are reported on the RHE scale.

The experiments conducted in HCO_3_^–^ showed a higher overall current density (Figure S7) and increased individual partial current densities for all products, HCOOH, CO, and H_2_. It is notable, though, that there is a higher impact on the H_2_ formation rate in the bicarbonate solution. As the potential is made more negative, the selectivity for HER is enhanced while decreasing the FE for CO and HCOOH at high overpotentials (<−1.0 V vs RHE) compared to sulfate electrolyte (Figure S8). At low overpotentials (>−0.9 V vs RHE), the HCO_3_^–^ pH 7 electrolyte is found to increase the FE for HCOOH. Similar observations have been made for flat Au electrodes under well-defined mass transport conditions,^29^ where HCO_3_^–^ has been found to increase the FE for CO at low negative potentials. However, at potentials greater than −0.9 V, HCO_3_^–^ becomes the dominant proton donor for HER, leading to a decline of the FE(HCOOH).

When examining the partial current densities employing K_2_SO_4_ and KHCO_3_ on a SHE scale (Figure S9d,e) using a constant cation concentration of [K^+^] = 0.1 and 1.0 mol L^–1^, we find that the reaction rates for HCOOH and CO formation are minimally affected by the anion identity and bulk pH. It is known that for reactions that the rate-limiting step involves an equal number of protons and electrons, the partial current density should not shift with changes in pH when plotted on a RHE scale. In contrast, if the rate-limiting step of the reaction does not involve a proton-coupled electron transfer, the partial current density should not shift with changes in the pH on the SHE scale.^37,38^ This result suggests that the step that limits the reaction rate does not involve the transfer of a proton but rather an electron transfer step. This finding is consistent with previous results that reported the CO formation on polycrystalline Au and CO and HCOO^–^ on polycrystalline Cu and Cu(100) formation is pH independent.^16,37,39^ These studies have shown that the formation of CO and HCOO^–^ is not affected by the composition and concentration of electrolyte anions since the buffering anions do not participate in the rate-limiting step of their formation.

In contrast, the HER is heavily affected by the bulk pH and the presence of buffering species HCO_3_^–^. We observed a significant increase in the HER rate in the presence of HCO_3_^–^ in the electrolyte. Specifically, in 1.0 mol L^–1^ KHCO_3_, the rate of HER was more than an order of magnitude higher compared to that of K_2_SO_4_ (Figure 3c). At [K^+^] = 0.10 mol L^–1^, the current density for HER is similar in K_2_SO_4_ and KHCO_3_ (Figure S9c), indicating that under these conditions the anion has no significant effect on the rate of HER. At high bicarbonate concentration, its contribution as a proton donor to HER is prominent, in agreement with previous studies that have shown that HCO_3_^–^ can act as a proton donor for HER on Au electrodes.^14,16,40^ The notable rise in activity for H_2_ in concentrated HCO_3_^–^ electrolytes suggests that the observed activity comes from the buffering ability of HCO_3_^–^ ions to directly transfer hydrogen atoms to the electrode surface for the HER, outcompeting water reduction. Additionally, when drastically increasing HER rates, one must be aware of possible cathodic corrosion effects. Studies by Sridhar et al.^41,42^ and Kyriacou et al.^43^ have reported that these effects are associated with the formation of tin hydride species at very negative potentials and high currents during long experiments, leading to electrocatalyst degradation and changes to the electrode surface, which are beyond the scope of this work.

## 4 Discussion

Based on the analysis of the partial current densities in sulfate and bicarbonate electrolytes on the SHE scale, we see that changes in pH and the anion did not affect the formation of HCOOH and CO, while the formation is strongly impacted by the concentration of the cation in solution. Similar to what has been previously highlighted and reported on Au and Cu surfaces,^16,37,39^ this finding suggests that the rate-limiting step for both HCOOH and CO formation comprises a cation-coupled electron transfer process rather than a concerted proton-coupled electron transfer.^44^ This is experimental evidence that the often proposed concerted proton-coupled electron transfer that leads directly to the formation of the *OCHO intermediate might not be the most adequate for explaining the formation of HCOOH under these conditions.

A recent work by Whittaker et al.^45^ employed computational work and infrared spectroscopy to identify and calculate reaction intermediates on Sn surfaces during CO_2_RR. By comparing the adsorption energies of carbon-bound *CO_2_^–^ and oxygen-bound *OCO^–^ intermediates, they concluded that the selectivity to HCOOH over CO on Sn surfaces cannot be rationalized in terms of the absorption energies of the different bound CO_2_^–^ intermediates. On the other hand, the formation of *H intermediate is expected at relevant potentials for CO_2_RR to HCOOH, as the adsorption energy of *H is more favorable than the adsorption of *CO_2_^–^. Although there is no direct spectroscopic evidence of hydrides in Sn for electrochemical CO_2_RR, hydride species on Sn-based catalysts have often been invoked in the CO_2_RR on molecular Sn-catalysis.^46,47^ The work of Sarma et al.^47^ employed density functional theory to study the hydride-assisted pathway on Sn-based metal oxide nanoparticles in the CO_2_RR, from which the negatively charged *H is identified as the species responsible for facilitating the formation of formate as a major product from CO_2_RR over CO.

Our experimental observations indicate that the formation of HCOOH is more strongly impacted by the cation than the CO formation, similar to what we have previously observed for a Pd-modified Pt catalyst, for which surface hydrides were also concluded to be the key catalytic intermediate in formate formation.^18^ This observation suggests that different intermediates are involved in the selectivity-determining step for the formation of HCOOH and CO. The difference in selectivity for HCOOH and CO, can be explained by the different key catalytic intermediates and their sensitivity to the effect of cations. While the formation of HCOOH likely occurs through the hydride-assisted pathway, the CO formation pathway is more likely to involve the negatively charged adsorbed *CO_2_^–^ intermediate, similar to the proposed mechanism for the CO formation on Au electrodes.^14,17^ The reaction rate for the formation of formic acid is governed by the surface coverage of *H, influenced by alkali cations that act as protons donor species in mildly acid media, as has been previously suggested.^48,49^ The effect of cations on the stabilization of this *CO_2_^–^ intermediate was found to be lower compared to the cation-induced stabilization of *H species, as calculated by density functional theory on the Pd-modified Pt(111) catalyst,^18^ which explains why the HCOOH production is more sensitive to the impact of cations than CO formation. By increasing the electric field, cations promote and maintain hydride formation at high *H coverage. The negatively charged hydride intermediate facilitates the formation of the formate (HCOO^–^), which is formed by a surface-catalyzed coupling of CO_2_ or *CO_2_ with *H^–^. This aligns with the recent literature proposal that surface hydride is the more plausible intermediate for HCOO^–^/COOH formation on Sn electrodes, while the *COOH leads to CO.^18,48^

We observed that the HER is sustained at low rates in mild acid media (pH 4), and this behavior remains largely unaffected by variations in the cation concentration, cation size, or applied potential within the studied conditions. At these conditions, the concentration of free protons is very low, and water reduction predominates as the primary reaction for H_2_ formation. Similarly, as recently reported by our group,^48^ on polycrystalline Au at pH 4 and in sulfate electrolytes, the water reduction reaction rate seems to be unaffected by electrolyte concentration, in agreement with what we observed for the H_2_ formation under CO_2_-saturated conditions. The observed differences in the effects of cations likely arise from their distinct roles in the mechanisms of reduction of CO_2_ to formic acid versus water reduction. In the first case, cations facilitate the formation of surface hydrides and their transfer to CO_2_. In contrast, water reduction on Sn appears to have a weaker cation dependence. Previous studies have highlighted the complex relationship between water reduction and local cation concentrations in Au and Pt surfaces in alkaline media,^50^ where water reduction predominates. On the other hand, in neutral media (pH 7) with high potassium bicarbonate concentration, there is a substantial promotion of HER. Also, cations likely enhance the reactivity of bicarbonate, as has been suggested for gold electrodes.^40^ Consequently, H_2_ formation is favored by the near-surface bicarbonate available as active proton donors. The considerably lower p*K*_a_ of bicarbonate compared to water highlights why it remains a principal source of protons, despite its lower concentration compared to water.^29,40^

## 5 Conclusions

In this work, we investigated how the electrolyte affects the selectivity and activity of the electrochemical reduction of CO_2_ on a metallic Sn electrode. We employed a two-compartment electrochemical cell to measure the amounts of HCOOH, CO, and H_2_ produced at different cathodic potentials and electrolyte conditions. At mild acid conditions (pH 4), we observed that at low overpotentials, when the reaction rates for HCOOH are still low, the dominant process is the HER, as evident from higher FE(H_2_), compared to FE(HCOOH) and FE(CO). As the overpotential increases, protons are progressively consumed, increasing the interfacial pH, and the CO_2_RR to HCOOH becomes the predominant reaction. The electrolyte concentration substantially influences the overall CO_2_RR activity, particularly for HCOOH production, while it has a lower impact on the CO rates. We identified a similar behavior for the effect of the cation identity with increasing cation size, in the order of Li^+^ < K^+^ < Cs^+^, which is more pronounced on the partial density currents of CO_2_RR to HCOOH. The rate of H_2_ production is hardly influenced by potential, electrolyte concentration, or cation size in pH 4.

Furthermore, we explored the impact of anions acting as proton donors, comparing sulfate and bicarbonate solutions while keeping the potassium ion concentration constant. The results revealed notable differences in activity, with higher overall current densities and increased partial current densities for the HER in concentrated bicarbonate solutions. This highlights the crucial role of anions as proton donors in affecting the activity of CO_2_RR on Sn electrodes and promoting the competitive HER. A high bicarbonate concentration (1.0 mol L^–1^) led to a significant increase in HER activity in neutral pH, drastically surpassing the HER activity at pH 4. At the same time, on the SHE scale, the reaction rate toward HCOOH and CO is not strongly influenced by the bulk pH and anion identity, but affected to different extents by the cation (concentration) in solution, which indicates that the reactions are limited by a cation-coupled electron transfer step. From these results, we have proposed that the formation of HCOOH on Sn electrodes proceeds through adsorbed hydrides that lead to the formation of the *OCHO intermediate, while the CO formation occurs through an electron transfer step forming the *CO_2_^δ−^ intermediate. We hypothesize that while the cations facilitate the formation of HCOOH and CO by stabilizing these negatively charged intermediates through electrostatic interactions, the difference in sensitivity of the promoting HCOOH and CO formation is related to the relative strength of the cation stabilizing effect on hydride species compared with *CO_2_^δ−^ intermediates.

Moreover, our study provides a comprehensive database of the activity and selectivity of Sn for the CO_2_RR in mild acidic media and highlights the importance of electrolyte engineering in HCOOH production on Sn electrodes. The choice of electrolyte can modulate the activity while maintaining high selectivity toward HCOOH and hindering HER. Although bicarbonate electrolytes are most often used, they may not always represent the best approach, especially at high concentrations, given their propensity to act as a proton donor and promote HER. In essence, although the overall activity increases, it does so at the expense of reducing the selectivity toward HCOOH, and the concentration employed should be carefully chosen. An alternative and potentially more effective strategy to enhance formic acid production could involve employing weakly solvated cations and elevating the cation concentration while utilizing nonprotic anions. Our insights presented here pave the way for optimizing reaction parameters to steer the CO_2_RR toward the desired outcome products, with a particular focus on formic acid.

## Supporting Information Available

Additional experimental results, including partial current densities for HCOOH, CO, and H_2_ for each electrolyte concentration, faradaic efficiencies for different alkali sulfates (Li, K^+^, and Cs^+^) and bicarbonate electrolyte (1.0 mol L^1^ KHCO_3_), total current densities for CO_2_RR and HER from −0.8 to −1.2 V vs RHE, and a comparison between currents for electrolytes containing sulfate and bicarbonate with constant K^+^ concentration on the RHE and SHE scales (PDF)
