# Supplementary material for: Electrolyte Effects on Electrochemical CO2 Reduction Reaction at Sn Metallic Electrode
Source: J Phys Chem C Nanomater Interfaces. 2024 Dec 5;128(50):21421–9. doi: 10.1021/acs.jpcc.4c06361 (PMC11664572; doi:10.1021/acs.jpcc.4c06361)
Supplement: Supplementary file 1 — jp4c06361_si_001.pdf [file jp4c06361_si_001.pdf]

**Supporting information to**

# Electrolyte Effects on Electrochemical $CO_2$ Reduction Reaction at Sn Metallic Electrode

Maria R. Pinto,[1,2] Rafaël E. Vos,[2] Raphael Nagao,[1,3] Marc T. M. Koper[2*]

[1] Institute of Chemistry, University of Campinas, Campinas, SP 13083-970, Brazil

[2] Leiden Institute of Chemistry, Leiden University, P.O. Box 9502, 2300 RA Leiden, The Netherlands

[3] Center for Innovation on New Energies, University of Campinas, Campinas, SP 13083-841, Brazil

E-mail: m.koper@lic.leidenuniv.nl

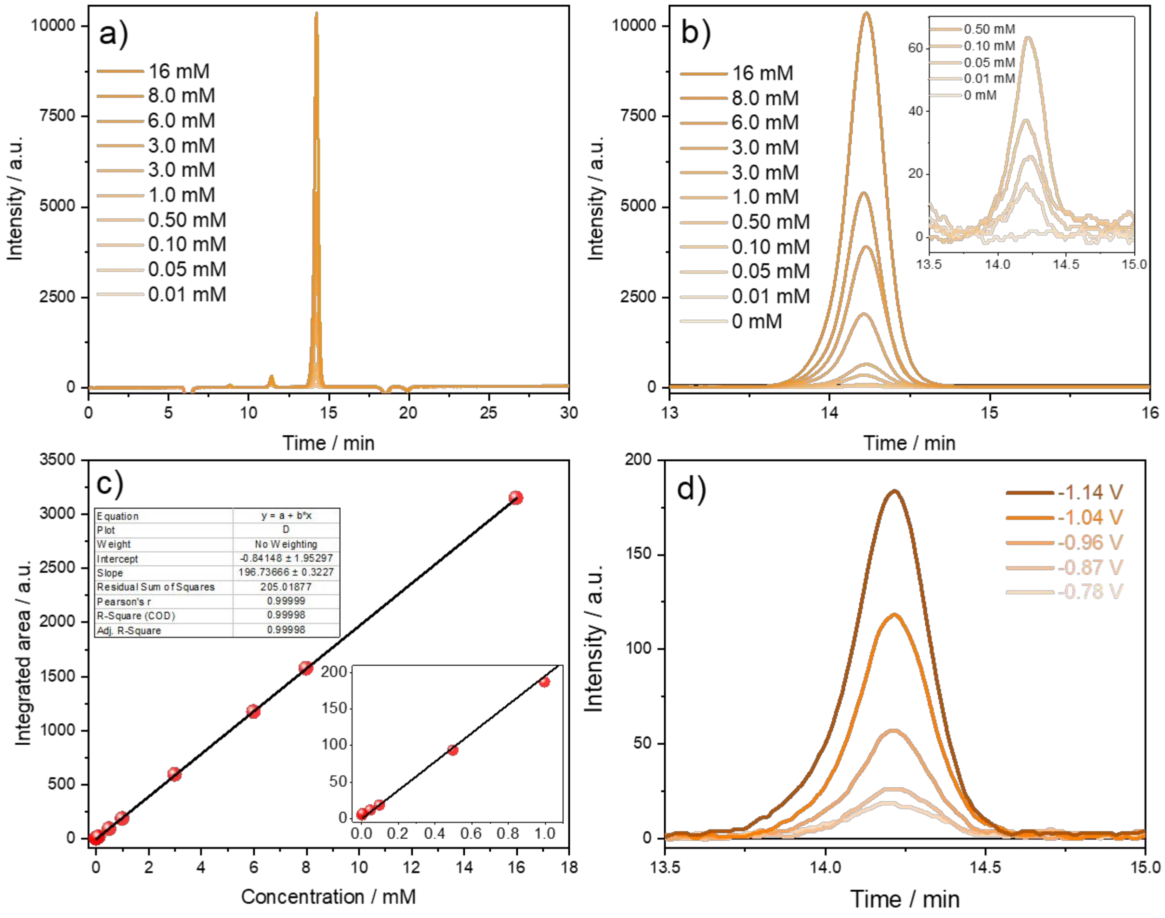


Figure S1: a) Chromatograms of standard samples, b) a zoomed-in view of the peak corresponding to formic acid, and c) the calibration curve for formic acid quantification. d) Zoomed-in chromatogram of the formic acid peak in the post-electrolysis electrolyte from experiments conducted at potentials as indicated in the image in [$K_2SO_4$] = 5 mM.

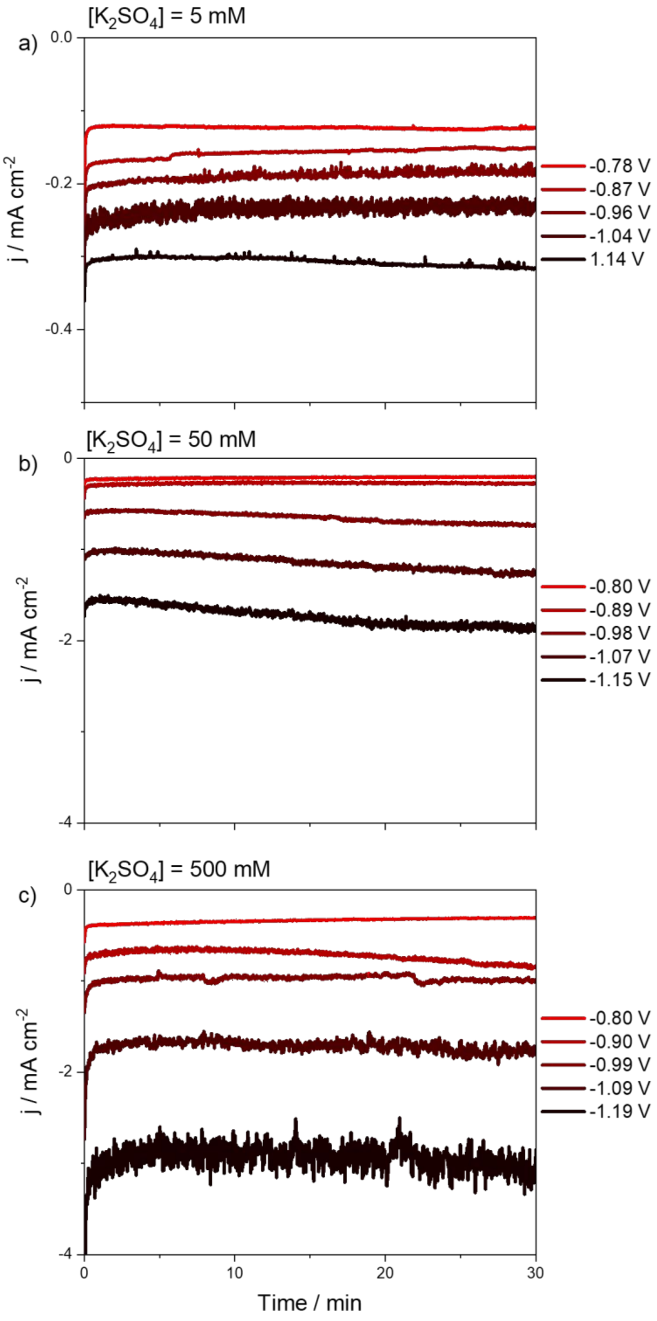


Figure S2: Samples of current density vs. time profile for potentiostatic electrolysis experiments over different potentials for $[K_2SO_4]$ = a) 5 mM, b) 50 mM, and c) 500 mM at fixed pH = 4. Potentials are referred vs. RHE scale.

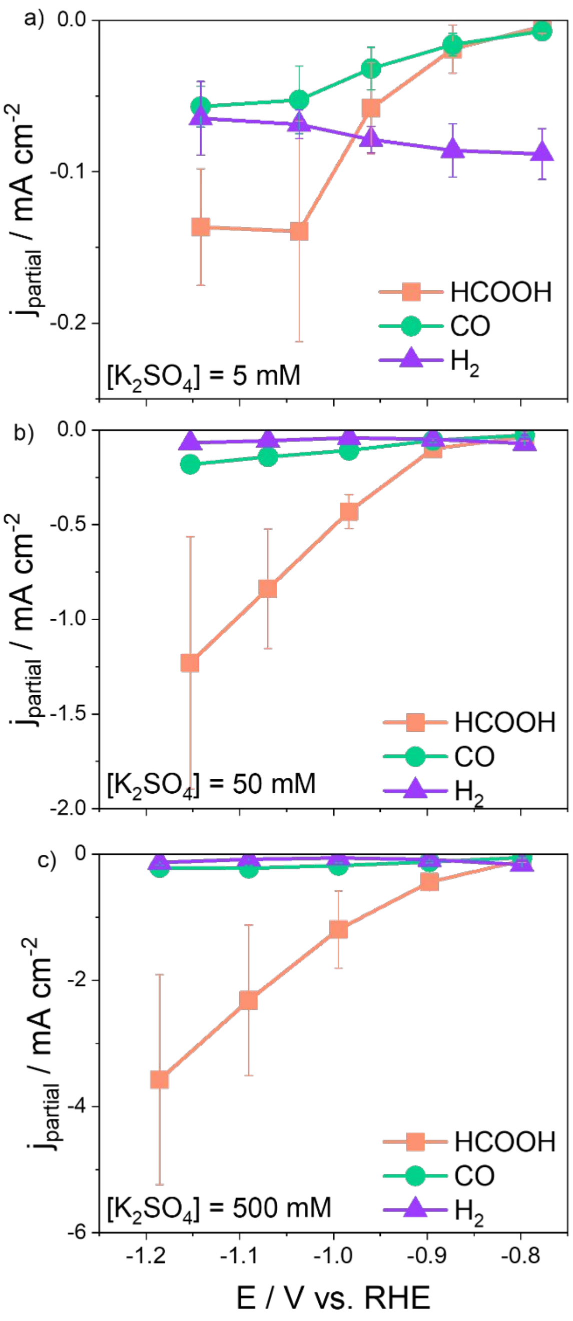


Figure S3: Partial current for HCOOH (orange), CO (green), and $H_2$ (purple) at different electrolyte concentrations at fixed pH 4. [$K_2SO_4$] = a) 0.01, b) 0.10, c) 1.00 mol $L^{-1}$. The error bars represented in the figure are twice the standard deviation, which are determined from at least three separate experiments. The lines are a guide to the eye.

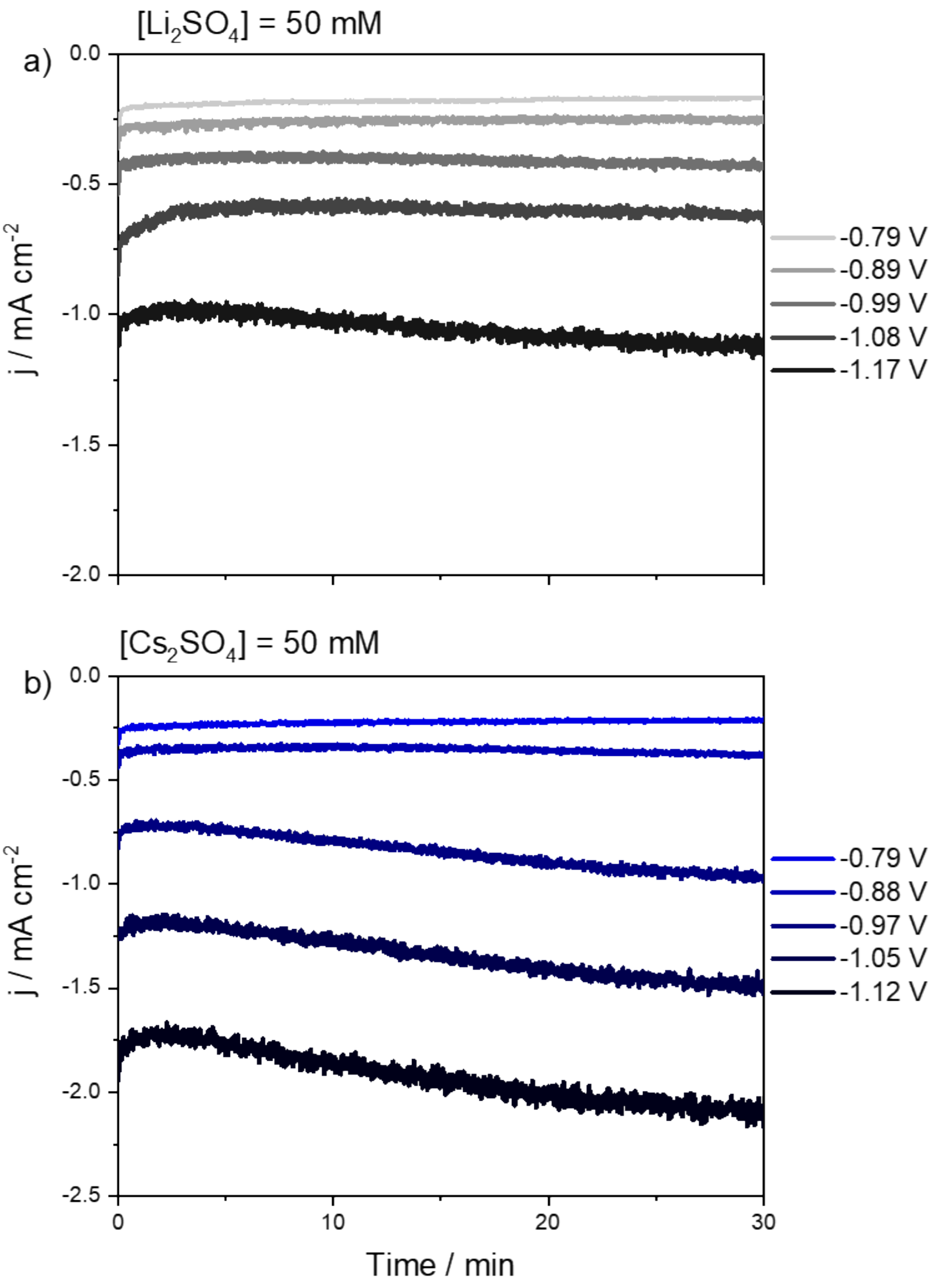


Figure S4: Samples of current density vs. time profile for potentiostatic electrolysis experiments over different potentials at pH 4 with different alkali sulfates, namely a) $Li^+$, and b) $Cs^+$ (blue) at 50 mmol $L^{-1}$. Potentials are referred vs. RHE scale.

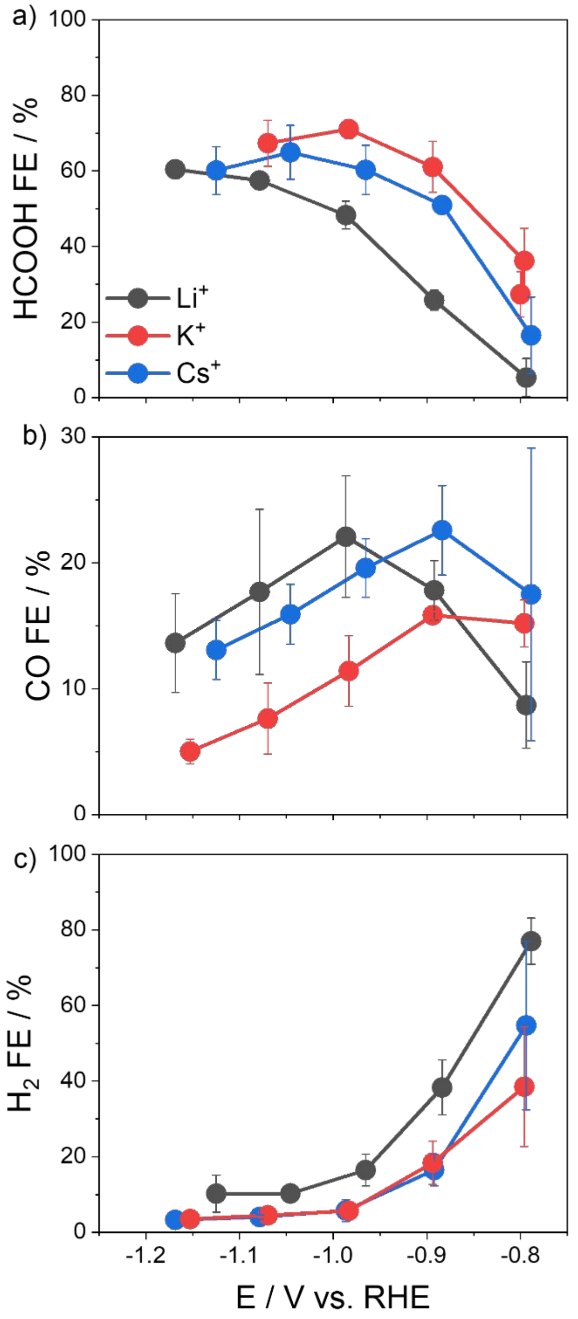


Figure S5: Selectivity for Sn at pH 4 at different alkali sulfates, namely $Li^+$ (black), $K^+$ (red), and $Cs^+$ (blue) at 50 mmol $L^{-1}$. Faradaic efficiencies for (a) formic acid, (b) carbon monoxide, and (c) hydrogen. The error bars represented in the figure are twice the standard deviation, which are determined from at least three separate experiments. The lines are a guide to the eye.

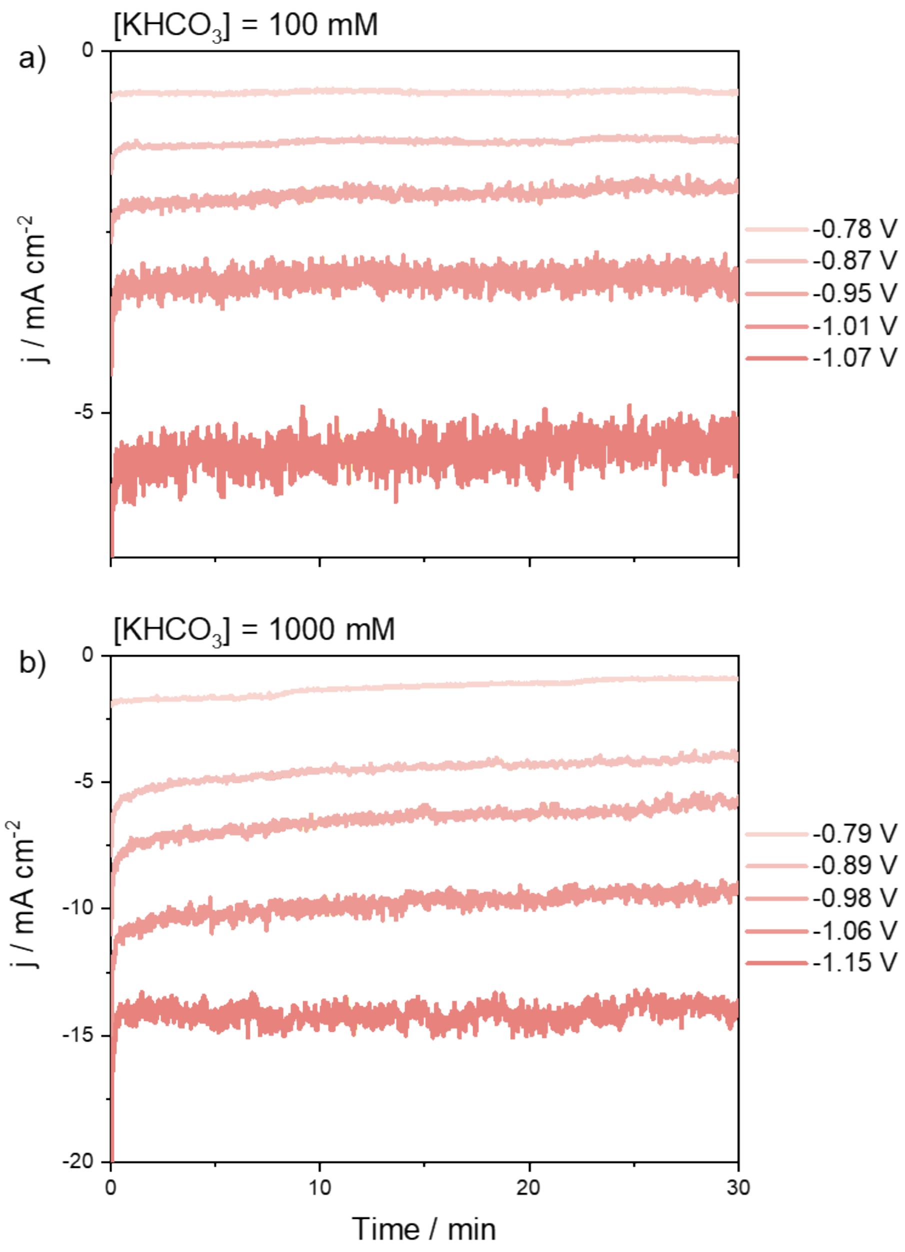


Figure S6: Sample of the current density vs. time profile for potentiostatic electrolysis experiments over different potentials at pH 7 and [$KHCO_3$] = 0.10 and 1.00 mol $L^{-1}$ pH 7. Potentials are referred vs. RHE scale.

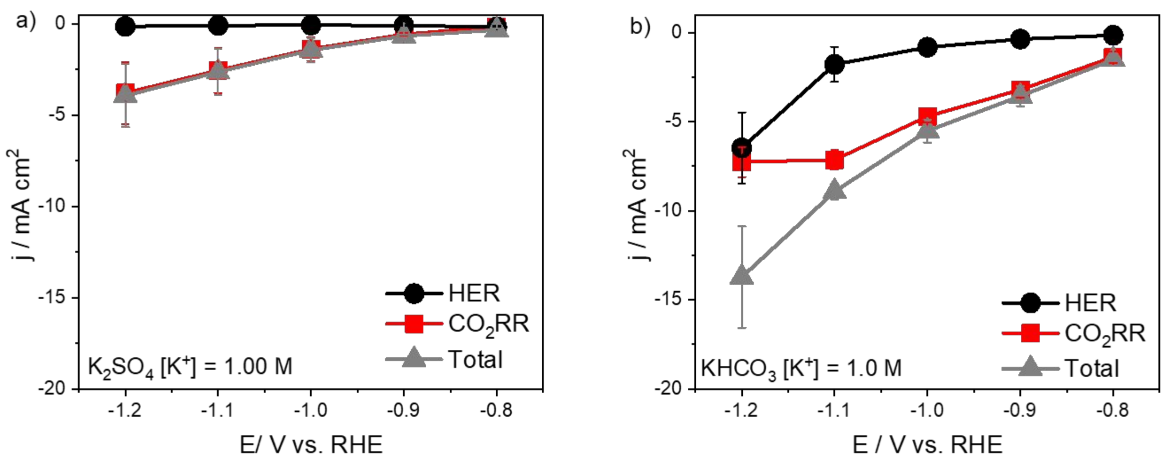


Figure S7: Current for HER (black), $CO_2$RR (red), and total current density (grey) in (a) [$K_2SO_4$] = 0.50 mol $L^{-1}$ pH 4 and (b) [$KHCO_3$] = 1.00 mol $L^{-1}$ pH 7 electrolytes. The error bars represented in the figure are twice the standard deviation, which are determined from at least three separate experiments. The lines are a guide to the eye.

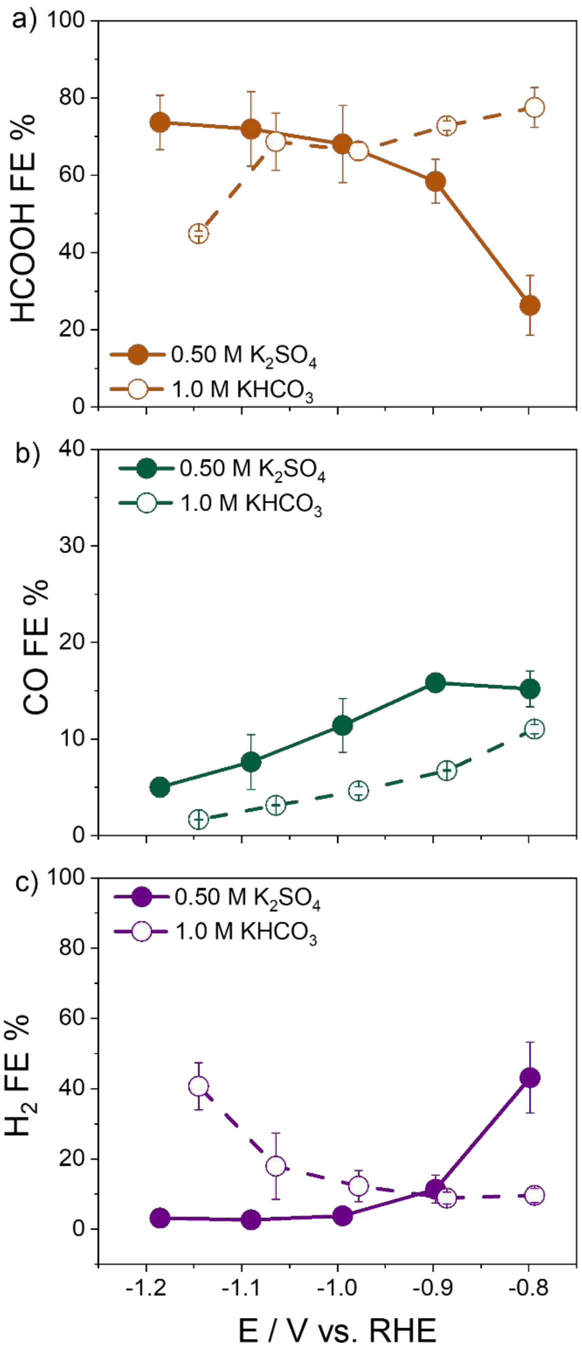


Figure S8: Faradaic efficiencies for (a) HCOOH (b) CO and (c) $H_2$ at [$K_2SO_4$] = 0.50 mol $L^{-1}$ pH 4 and [$KHCO_3$] = 1.00 mol $L^{-1}$ pH 7 electrolytes. The error bars represented in the figure are twice the standard deviation, determined from at least three separate experiments. The lines are a guide to the eye.

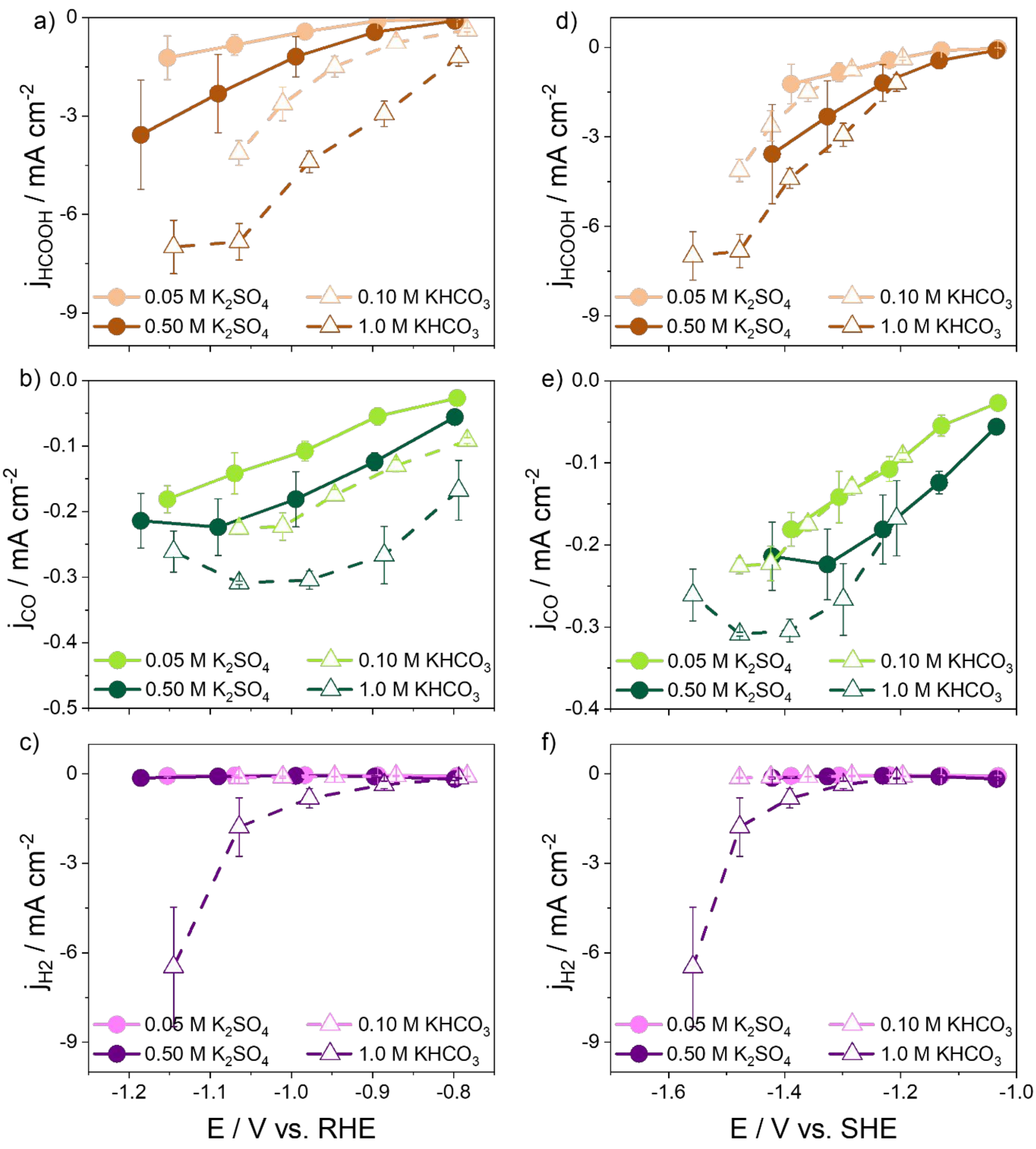


Figure S9: Partial current for (a, d) HCOOH, (b, e) CO, and (c, f) $H_2$ in $[K_2SO_4]$ = 0.05 and 0.50 mol $L^{-1}$ pH 4 and $[KHCO_3]$ = 0.10 and 1.00 mol $L^{-1}$ pH 7 in RHE scale (a-c) and SHE scale (d-f). The error bars represented in the figure are twice the standard deviation, which are determined from at least three separate experiments. The lines are a guide to the eye.
